# Practical Notes and Formulæ

**Published:** 1882-08-20

**Authors:** 


### PRACTICAL NOTES AND FORMULÆ.

**In Dyspepsia with Acid Eructations and Debility**—

℞ Ammoni. carbonat.......................... grs. 5,

Tinct. aurantii........................... ʒ 1,

Infus. chirtteæ........................... ℥ 1,

Aquæ.................................. ad ℥ 2.

Make a draught, to be taken night and morning.—*Western Med. Reporter.*

Or:

℞ Sodæ bicarbonat.......................... grs. 120,

Spts. ammon. aromat....................... ʒ 1,

Tinct. zingiberis......................... ℥ 1,

Infus. gent. co........................ad ℥ 8.

Mix. A sixth part three times a day.—*Ibid.*

**In Pyrosis and Gastrodynia.**—

℞ Liq. bismuthi et ammon. citrat................. ʒ 1,

Infus. quassiæ.............................. ℥ 1.

Make a draught, to be taken three times a day. One drachm of the solution of bismuth is equal to 20 grains of powder.—*Ibid.*

**Camphorated Chloro-Tannate of Iodine** is the name given by Dr. Q. C. Smith, of Austin, Texas, to the following preparation which is used as a topical application to bleeding ulcers and cancers ot the cervix uteri:

℞ Chloral hydrate..........................ʒ i,

Iodine.................................ʒ ss,

Oil of Camphor..........................ʒ vi.

Dissolve and add sufficient tannic acid to bring the mixture to the consistence of thick syrup.—*South. Prac.*

**Injection for Sciatica.**—Lereboullet recommends in cases where morphia is badly borne the following solution for hypodermic injection—

℞ Morphiæ hydrochlor........... gr. ½, 0.03 Gm.,

Atropiæ sulph................ gr. ⅙, 0.012 Gm.,

Aquæ destillat................ fl. ʒijss, 10.00 fl. Gm.

Ten to fifteen minims every six hours.—*Union Med.; London Pract.*

**Treatment of Phagedenic Ulcers.**—Dr. Vidal, in Concours Medical, recommends—

℞ Vaseline................................ ʒ x,

Pyrogallic acid.......................... ʒ j.

M. Make into an ointment and apply morning and evening.—*Med. and Surg. Rep.*

**Incontinence of Urine.**—For incontinence of urine in children, Dr. Janeway, in N. Y. Medical Record, recommends a combination of ergot, belladonna and iodide of iron. We suggest the following formula—

℞ Tinct. ergot ........ ʒ ij,

Tinct. belladonna ........ ʒ j,

Syr. iodide iron ........ ℥ j,

Simple elixir ........ ʒ j.

M. One teaspoonful morning, noon and bedtime to a child 10 years old.

**In Heart-burn and Acid Eructations.**—The following lozenges are superior to the officinal bismuth lozenges—

℞ Bismuth subnitrat ........ grs. 720,

Magnesiæ carbonat ........ ℥ 2,

Calcis carbonat. præcipitat ........ ℥ 3,

Sodæ bicarbonat ........ grs. 1800,

Sacchari albi ........ ℥ 14,

Acaciæ gummi ........ grs. 220,

Mucilag. acaciæ ........ ℥ 1.

Divide into 360 lozenges, and dry them with a moderate heat. From one to six lozenges may be taken at a dose.—*Ibid.*

**Iodoform in Uclers.**—Dr. Wade, in Detroit Clinic, says: In the treatment of ulcers, the cause, if possible, being otherwise removed, I have found no other application to equal the following:

Take of

Iodoform, powdered ........ grs. 30,
Sub-nitrate of bismuth ........ grs. 60,
Chloral hydrate ........ grs. 15,
Glycerine ........ ℥ 2,
Oil of rose geranium ........ gtt. 10,
Water to make ........ fl. ʒ 3.

Mix and write. Shake and apply.

**Purpura.**—

℞ Vin. ferri ........ ℥ iv,

Liq. arsenicalis ........ *m* xx,

Sry. zingiberis, ........ ℥ ij.

M. Sig. One-sixth part, with three tablespoonfuls of water three times a day, after meals.—*Med. Gaz.*

**Dyspnœa of Phthisis and Emphysema.**—

℞ Ext. stramonii ........ grs. iij,

Ext. hyoscyami ........ grs. xx,

Ext. lupuli ........ grs. xl.

M. and divide into twelve pills; one to be taken every four hours until relief is obtained.—*Med. Gaz.*

**Iodoform in Lung Disease.**—The recent developments in the pathology of lung infiltration, and in its antiseptic treatment by continuous inhalations, have given me hope to expect that the continuous action of so potent a local remedy as iodoform, when precipitated upon the surface of the minute ramifications of the lungs, may develop more than has recently been anticipated to accrue in the local treatment of consumption. I have not yet had an opportunity of furnishing clinical evidence in support of this theory, and I bring up the subject in the interests of a cooperative test. This is the formula I use:

Take of

Acetic ether..............................fl. ℥ 4,

Iodoform..............................grs. 5,

Glycerine..............................fl. ℥ 4,

Chloral hydrate..............................grs. 5 to 10.

Mix. Dissolve the iodoform before adding the glycerine. The acetic ether should be chemically pure, which may not be found in the shops. I have not had a satisfactory article, until it was made with reference to purity by Swift & Dodds, of Detroit, especially for this purpose. Some samples would not mix with glycerine, and the impure goods rapidly change the iodoform to free iodine. Acetic ether quite effectually covers the odor of iodoform, at least so that the inhalation is not at all disagreeable. I have now used iodoform in this manner several hundred times, and I have not yet observed any undesirable effects, either local or constitutional.—*Dr. Wade in Detroit Clin.*

**Prevention of Diptheritic Infection.**—M. Hager, in the Pharm. Centralblatt., recommends lozenges, composed after the following formula, to persons who are exposed to the infection of diphtheria:

℞ Cetæ alb. (white wax)..............................ʒ v,

Colophon. (rosin)..............................ʒ iss.

M. Melt together, and then add—

℞ Tolutan. bals..............................ʒ iiss,

Pulv. aromat..............................ʒ iss,

Sacch. alb..............................ʒ v,

Ac. benzoic..............................ʒ iss–iiss.

M. Reduce to fine powder, and aromatize with—

℞ Ol. cinnamomi..............................*m* x,
Creasot..............................ʒ j.

M. After the mass has cooled divide into 100 pastilles.

One of these lozenges should be very slowly masticated four or five times daily.—*Med. and Surg. Rep.*

